# Combined application of biochar and nitrogen fertilizer promotes the activity of starch metabolism enzymes and the expression of related genes in rice in a dual cropping system

**DOI:** 10.1186/s12870-021-03384-w

**Published:** 2021-12-18

**Authors:** Izhar Ali, Saif Ullah, Anas Iqbal, Zhao Quan, He Liang, Shakeel Ahmad, Ihsan Muhammad, Amanullah, Imran, Zixiong Guo, Shangqing Wei, Ligeng Jiang

**Affiliations:** 1 College of Agriculture, Guangxi University, Nanning, 530004 Guangxi China; 2 College of Life Science and Technology, Guangxi University, Nanning, China; 3 Department of Agronomy, Faculty of Crop Production Sciences, University of Agriculture, Peshawar, Pakistan

**Keywords:** Rice, Biochar, Starch-related genes, Starch synthesis enzyme, Amylose content, Grain yield

## Abstract

**Background:**

Overuse of chemical fertilizer highly influences grain filling rate and quality of rice grain. Biochar is well known for improving plant growth and grain yield under lower chemical fertilization. Therefore field trials were conducted in the early and late seasons of 2019 at Guangxi University, China to investigate the effects of combined biochar (B) and nitrogen (N) application on rice yield and yield components. There were a total of eight treatments: N1B0, 135 kg N ha^− 1^+ 0 t B ha^− 1^; N2B0,180 kg N ha^− 1^+ 0 t B ha^− 1^; N1B1,135 kg N ha^− 1^+ 10 t B ha^− 1^; N1B2,135kg N ha^− 1^+ 20 t B ha^− 1^; N1B3,135 kg N ha^− 1^+ 30 t B ha^− 1^; N2B1,180 kg N ha^− 1^+ 10 t B ha^− 1^; N2B2,180 kg N ha^− 1^+ 20 t B ha^− 1^; and N2B3,180 kg N ha^− 1^+ 30 t B ha^− 1^.

**Results:**

Biochar application at 30 t ha^− 1^combined with low N application (135 kg ha^− 1^) increased the activity of starch-metabolizing enzymes (SMEs) during the early and late seasons compared with treatments without biochar. The grain yield, amylose concentration, and starch content of rice were increased in plots treated with 30 t B ha^−1^and low N. RT-qPCR analysis showed that biochar addition combined with N fertilizer application increased the expression of *AGPS2b*, *SSS1*, *GBSS1*, and *GBSE11b*, which increased the activity of SMEs during the grain-filling period.

**Conclusion:**

Our results suggest that the use of 20 to 30 t B ha^− 1^coupled with 135 kg N ha^− 1^ is optimal for improving the grain yield and quality of rice.

**Supplementary Information:**

The online version contains supplementary material available at 10.1186/s12870-021-03384-w.

## Background

Rice (*Oryza sativa* L.) is a major dietary energy source (30–60% of the calories in the diet) and staple food for more than 4 billion people in Asia [1–3]. Approximately 70 to 90% of the dry weight of the starch in rice endosperm is composed of amylose and amylopectin. The ratio of amylose and amylopectin is often measured to evaluate the physicochemical characteristics and nutritional quality of rice [1]. Starch metabolism enzymes (SMEs) promote the accumulation of amylose and amylopectin and include ADP-glucose pyrophosphorylase (AGPase), granule-bound starch synthase (GBBS), starch branching enzyme (SBE), starch debranching enzyme (DBE), soluble starch synthase (SSS), and starch synthase (SS) [1, 4, 5]. These enzymes are strongly positively associated with amylose content and starch composition [1, 6]. Monitoring the activity of these SMEs and the expression of related genes under biochar application combined with nitrogen (N) fertilizer could provide insight into the accumulation of starch and amylose in rice during the grain-filling stage. However, previously it is documented that sole biochar amendments improved on rice starch properties [7], starch metabolism enzymes under pot experiment [8], and improved transcript levels of genes encoding starch synthases [7]. But information on the effects of biochar application along with different N rates on SMEs, related genes expressions, yield and its relationship to starch and amylase content are not well reported.

Interest in biochar application as a soil amendment for sustainable agriculture has significantly increased in recent decades. Biochar is a carbonaceous material made from the incomplete combustion of different organic materials, such as plant straw, and it has been widely reported to stimulate plant growth and development [9]. It is believed that biochar has a long average dwelling time in soil, ranging from 1000 to 10,000 years, with an average of 5000 years [10–12]. Therefore, one time use of biochar application for long term outputs in sustainable agriculture is economically feasible and can be adopted on a mass scale [13]. Previously it is documented that biochar combined with N fertilizer increases plant productivity [14], reduces greenhouse gas emissions [15], enhances soil physiochemical properties, and promotes the growth of beneficial soil biota [8]. Biochar is a soil conditioner that promotes soil microbial activity and improves grain quality [9], improves soil N accessibility and retention, decreases soil bulk density, improves the water-holding capacity of soil, increases the pH and cation exchange capacity, increases the abundance of beneficial microorganisms, and reduces the bioavailability of heavy metals, all of which improve plant photosynthesis [16, 17]. Gong et al. [7] reported that sole biochar promoted SMEs, related genes expression and starch properties in pot experiment. Furthermore, our previous one season pot experiment results showed that biochar plus N fertilizer enhanced SMEs and starch content as compared to sole N applied treatment [14]. More research is needed under field condition to explore the ability of biochar application combined with inorganic fertilizer to promote the activity of SMEs, increase the expression of related genes, and increase the starch and amylose content of rice.

Previous studies examining the expression of genes in plants treated with biochar have shown that biochar application can promote plant growth [18]. Starch-metabolizing genes are expressed up regulated in transgenic maize [19, 20], rice leaves [20], potatoes [21], and grapes [22] in response to N fertilizers as compared to control treatments. Furthermore, several studies have examined the effect of temperature and organic manure on the activity of starch biosynthesis-related enzymes [1, 23]. However, few studies have characterized the activity of SMEs and the expression of related genes in noodle rice grain in response to combined biochar and N fertilizer application under pot experiment [8]. The objectives of this study were to (1) characterize the effect of combined biochar and synthetic N fertilizer application on the growth, yield, and yield components of rice; (2) assess the effect of combined biochar and synthetic N fertilizer application on the activity of grain SMEs and the expression of related genes; and (3) investigate the effect of combined biochar and N fertilizer application on the starch and amylose content of rice grains under field condition.

## Materials and methods

### Biochar, soil, and field experimental setup

Field experiments were conducted at an experimental farm at Guangxi University during the spring (March–June) and fall (August–November) of 2020. The farm is characterized by a subtropical monsoon climate and an average annual rainfall of 1080 mm. The average minimum and maximum temperature and the soil physiochemical properties of the experimental site are presented in our previous studies [17]. Biochar was produced from cassava straw. The straw was burned in a traditional kiln (thermally insulated chamber, a type of oven) initially for 30 min in the absence of oxygen. Subsequently, pyrolysis was conducted for 96 h at about 500 °C. The physiochemical properties of biochar are presented in our previous studies [17, 24]. Experiments were conducted using a randomized complete block design with three replications, a plot size of 3.9 m × 6 m (23.4m^2^), and eight treatments (biochar = B): (1) N1B0, 135 kg N ha^− 1^+ 0 t B ha^− 1^; (2) N2B0, 180 kg N ha^− 1^+ 0 t B ha^− 1^; (3) N1B1, 135 kg N ha^− 1^+ 10 t B ha^− 1^; (4) N1B2, 135 kg N ha^− 1^+ 20 t B ha^− 1^; (5) N1B3, 135 kg N ha^− 1^+ 30 t B ha^− 1^; (6) N2B1, 180 kg N ha^− 1^+ 10 t B ha^− 1^; (7) N2B2, 180 kg N ha^− 1^+ 20 t B ha^− 1^; and (8) N2B3, 180 kg N ha^− 1^+ 30 t B ha^− 1^. The rice cultivar “Zhenguiai” was used as the test crop, which is the most commonly grown variety in Southern China. We bought it from the registered company CP seed industry Yunnan Zhengda seed Co. Ltd., China. Uniform seedlings (25 days old) were transplanted in the field, and two seedlings per hill were planted. The recommended doses of phosphorous (P, 75 kg ha^− 1^) and potassium (K, 150 kg ha^− 1^) (equaling 930 g P plot^− 1^ and 605 g K plot^− 1^, respectively) were used. Urea was applied in three split doses: 50% as a basal dose, 30% at the early tillering stage, and 20% at the panicle initiation stage; K was applied in two splits: 50% as a basal dose and 50% at the tillering stage. P and biochar were applied as a basal dose 1 day before transplanting (Table 1). Agronomic practices (e.g., irrigation, pesticide, and herbicide application) were the same across all experiments.

**Table 1:** The amount of N and biochar applied for each treatment at different time points

Treatment	(N kg ha^− 1^)	Biochar (t ha^− 1^)	Biochar (kg plot^− 1^)	Basal fertilization (g plot^− 1^)	Tillering (g plot^− 1^)	Panicle initiation (g plot^− 1^)
N1B0	135	0	0	Urea: 343.36 P_2_O_5_: 930 KCl:305	Urea:206.02 KCl: 305	Urea: 137.3
N2B0	180	0	0	Urea: 457.82 P_2_O_5_: 930 KCl:305	Urea: 274.69 KCl: 305	Urea: 183.13
N1B1	135	10	23.4	Urea: 343.36 P_2_O_5_: 930 KCl:305	Urea:206.02 KCl: 305	Urea: 137.3
N1B2	135	20	46.8	Urea: 343.36 P_2_O_5_: 930 KCl:305	Urea:206.02 KCl: 305	Urea: 137.3
N1B3	135	30	70.2	Urea: 343.36 P_2_O_5_: 930 KCl:305	Urea:206.02 KCl: 305	Urea: 137.3
N2B1	180	10	23.4	Urea: 457.82 P_2_O_5_: 930 KCl:305	Urea: 274.69 KCl: 305	Urea: 183.13
N2B2	180	20	46.8	Urea: 457.82 P_2_O_5_: 930 KCl:305	Urea: 274.69 KCl: 305	Urea: 183.13
N2B3	180	30	70.2	Urea: 457.82 P_2_O_5_: 930 KCl:305	Urea: 274.69 KCl: 305	Urea: 183.13

### Soil and biochar analysis

The physiochemical properties of the field site were assessed prior to the experiments. Soil organic carbon was determined by oxidizing 0.5 g of soil samples with 1 M K_2_Cr_2_O_7_-H_2_SO_4_and 5 mL of concentrated H_2_SO_4_, boiling at 170 °C for 6 min, and finally titrating the samples with FeSO4 [25, 26]. To determine the total N content, 200 g of soil was digested using the salicylic acid-sulfuric acid method of [27]. Total N was determined using the micro-Kjeldhal method [28]. The total N and K contents were obtained using the procedure described by Murphy & Riley [29], and Lierop & Gough [30], respectively.

### Expression analysis of starch metabolism-related isoform genes

#### Total RNA extraction and qRT-PCR

Grain samples of rice were taken from each treatment at 7, 14, 21, and 28 days after anthesis during both early and late seasons in liquid N and storedat–80 °C for RNA extraction. A Pure plant Kit 155 (TIANGEN, 432, Beijing, China) was used to obtain RNA. First-strand cDNA synthesis was conducted using 1 μg of RNA HiScript III-RT SuperMix for qPCR (gDNA wiper) per the manufacturer’s instructions (Vazyme, R323-01, Nanjing, China). qRT-PCR was conducted in a total volume of 20 μL containing 10 μLof Cham QTM Universal SYBR qPCR Master Mix (Q711-02/03, Nanning, China), 0.4 μL of both forward and reverse primer (10 mM), 1 μLof diluted cDNA, and 8.2 μLof RNase-Free ddH_2_0. Each step was performed with three biological replicates in the lab. Detailed information on primer pairs for gene evaluation is shown in the supplementary file named “Supplementary file-1 Table 1”. Expression of the *actin* gene (LOC-4333919) (used as a reference internal control gene) was used to normalize the expression of the amplified genes, and the relative expression of these genes was determined used the 2-^ΔΔCT^ method [31].

#### Starch metabolism enzymes activities (SMEs)

SMEs were extracted using the method of Nakamura et al. [32]. First, frozen grain samples were crushed using a pestle and mortar containing 5 mL of buffer solution. The buffer solution (pH 7.5) contained 100 mM HEPES-NaOH, 8 mM MgCL_2_, 2 mmol/L EDTA, 50 mM 2-mercaptoethanol, glycerol (v/v) 12.5, and 5% insoluble polyvinylpyrrolidone-40 (w/v). Finally, 30 μL of homogenate with 1.8 mL of buffer solution was balanced in 2 mL of buffer solution to determine GBSS activity. The remaining homogenate solution was centrifuged at 1000 rpm for 5 min at 0–4 °C, and the resulting supernatant was used for AGPase, SSS, SBE, and DBE assays by following the method of Nakamura et al. [32].

### Starch, amylose, and amylopectin measurements

The content of starch, amylose, and amylopectin was calculated using the dual-wavelength iodine binding method [33, 34]. First, rice was ground using a mortar, and powder was degreased twice with anhydrous ether. A 100-mg fraction of each sample was used to measure the amylose content and amylopectin content. A calibration curve was derived using pure amylose and amylopectin from potato (A0512, Sigma-Aldrich, St. Louis, MO, USA, and A8515, Sigma-Aldrich, respectively). Total starch content was calculated as the sum of the content of amylose and amylopectin.

### Statistical analysis

Data were analyzed using ANOVA in Statistics 8.1 software. Differences in means were assessed using the least significant difference (LSD) test (*p* < 0.05). Relationships between yield traits and SMEs were assessed using Pearson correlation analysis, and R software was used to construct the heat map.

## Results

### Effect of different levels of biochar and N on the activity of SMEs

#### SS activity

**Fig. 1:**
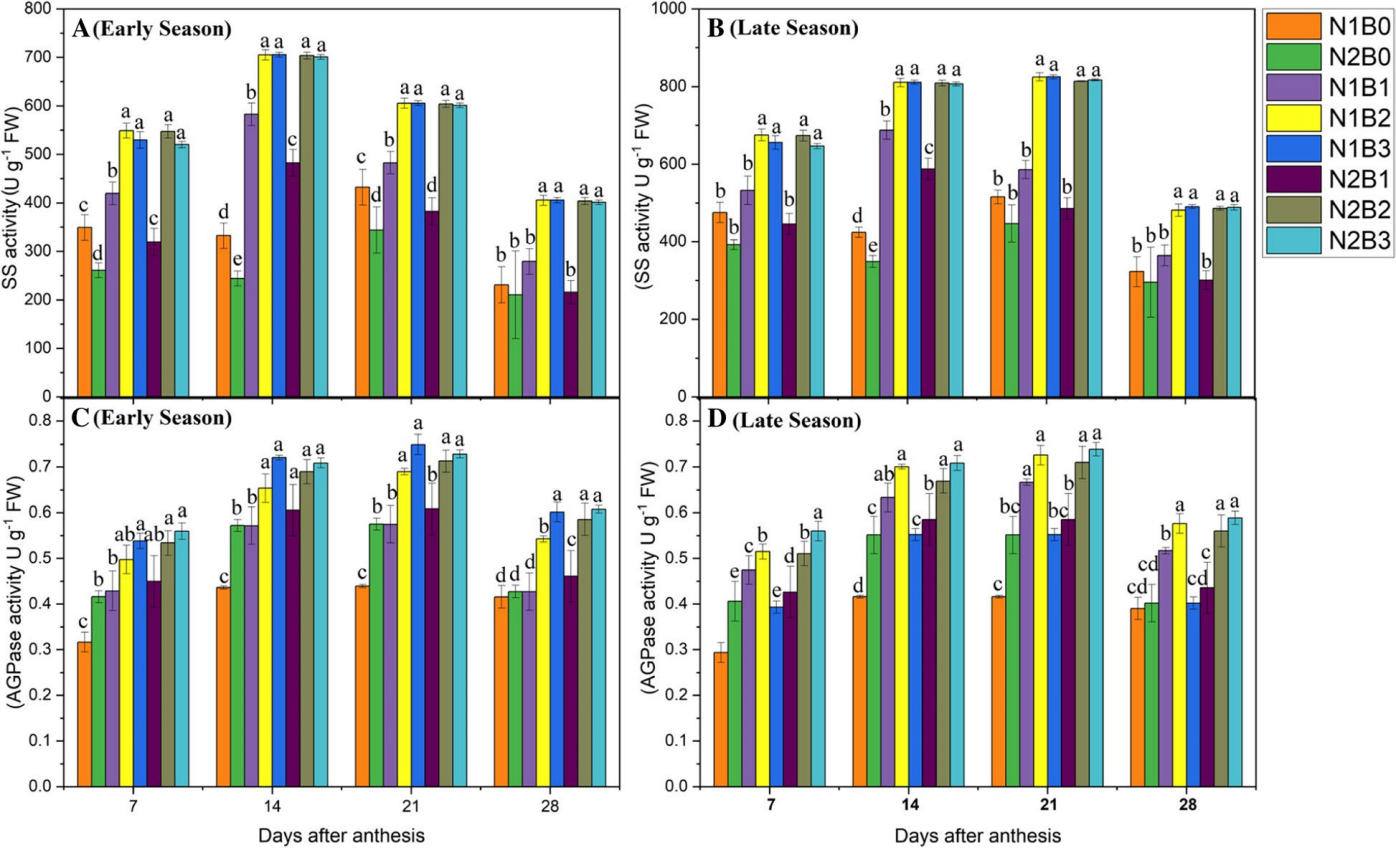
Changes in SS and AGPase activity 7, 14, 21, and 28 days after anthesis in response to different levels of biochar and N application in the early (**A**, **B**) and late(**C**, **D**) seasons. Vertical bars indicate the standard error of the mean. Different letters above columns indicate significant differences (*P* < 0.05). Vertical bars represent the standard error of the mean. Note: N1B0–N, lower dose + Control (no biochar); N2B0–N, higher dose + Control (no biochar); N1B1–N, lower dose + Biochar 10 t ha^− 1^; N1B2–N,lower dose + Biochar 20 t ha^− 1^; N1B3–N, lower dose + Biochar 30 t ha^− 1^; N2B1– N, higher dose + Biochar 10 t ha^− 1^; N2B2– N, higher dose + Biochar 20 t ha^− 1^; and N2B3– N, higher dose + Biochar 30 t ha^− 1^

In the cytoplasm of endosperm cells, SS breaks sucrose into uridine diphosphate glucose and fructose. In the present study, biochar application combined with nitrogen fertilizer significantly (*P* < 0.05) affected SS activity during both season and regimes (Supplementary file 2- Table 1)*.* SS activity was higher at 21 days after anthesis (DAA), followed by 14, 7, and 28 DAA (Fig. 1A, B). SS activity increased gradually from 7 to 21DAA and then decreased after 21DAA. The patterns of SS activity among treatments were the same during both early and late seasons. SS activity was 37, 35, and 31% higher in N1B2, N2B2, and N1B3 compared with N1B0 and 64, 54, and 59% higher compared with N2B0, respectively, during both seasons. SS activity under the lowest rate of N application (N1) was 14% greater compared with SS activity under the highest N rate across biochar treatments. No significant differences in SS activity were observed among N1B2, N1B3, N2B2, and N2B3. SS activity was the lowest in N2B0.

#### ADGPase activity

During the grain-filling stage, ADPase activity gradually increased up to 21 DAA and decreased thereafter until 28 DAA (Fig. 1C, D). Our results showed biochar in combination with nitrogen fertilizer considerably (*P* < 0.05) effected ADGPase activity (Supplementary file 2- Table 2). The same patterns of ADPase activity were observed among treatments during both early and late seasons. ADPase activity was 66, 64, and 59% higher in N2B3, N1B3, and N2B2 compared with N1B0 and 33, 31, and 27% higher compared with N2B0, respectively, in both seasons. ADPase activity was 5% higher at a high N rate compared with a low N rate across all biochar treatments. The lowest ADPase activity was observed in treatments in which biochar was not applied. No significant differences in ADPase activity during the grain-filling period were observed among N2B3, N1B3, and N2B2.

#### SBE and DBE activity

**Fig. 2:**
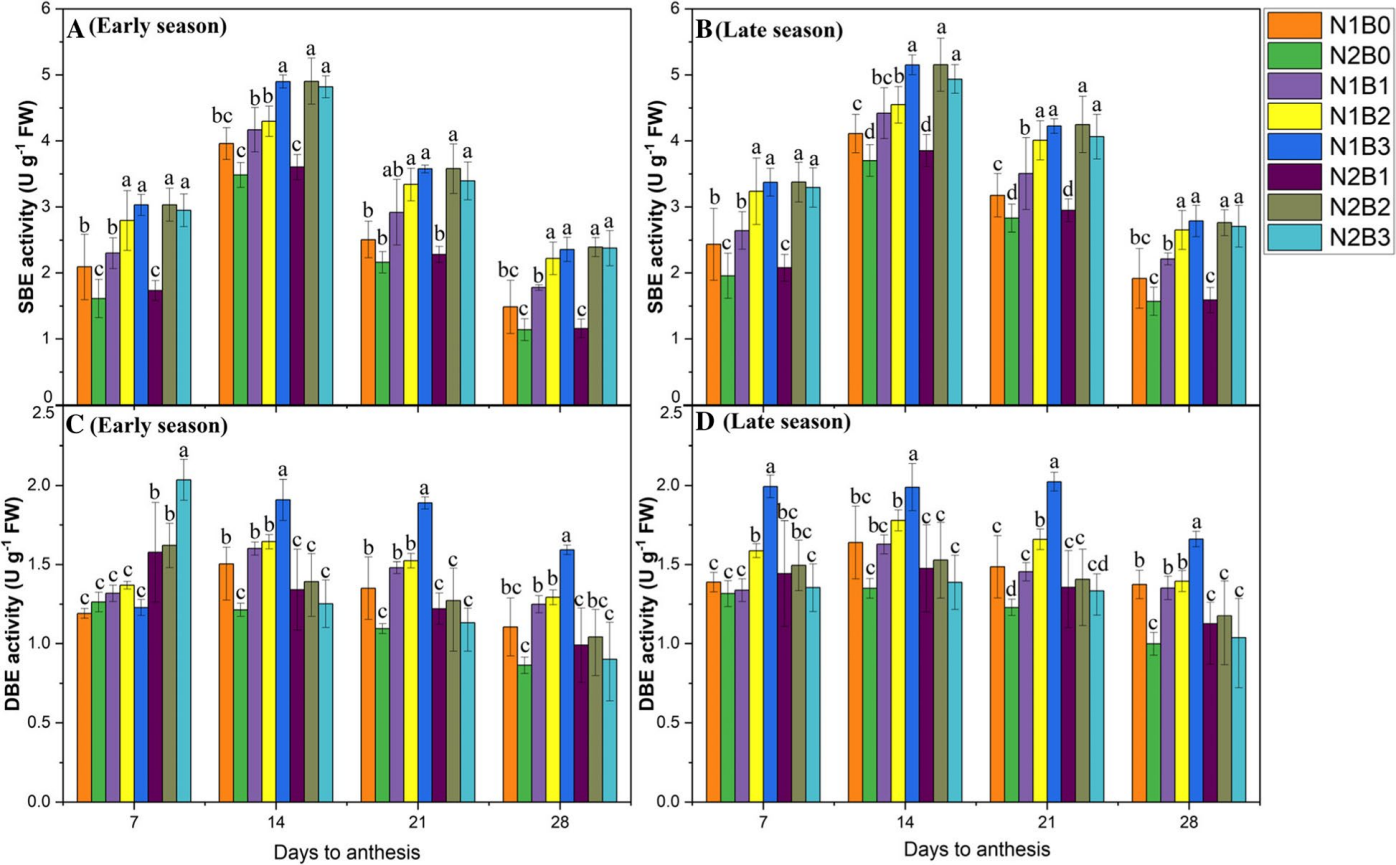
Changes in SBE and DBE activity 7, 14 21, and 28 days after anthesis in response to different biochar and N levels in the early (**A**, **C**) and late (**B**, **D**) seasons. Vertical bars represent the standard error of the mean. Different letters above columns indicate significant differences (*P* < 0.05). Vertical bars represent the standard error of the mean. Note: N1B0–N, lower dose + Control (no biochar); N2B0–N, higher dose + Control (no biochar); N1B1–N, lower dose + Biochar 10 t ha^− 1^; N1B2–N,lower dose + Biochar 20 t ha^− 1^; N1B3–N, lower dose + Biochar 30 t ha^− 1^; N2B1– N, higher dose + Biochar 10 t ha^− 1^; N2B2– N, higher dose + Biochar 20 t ha^− 1^; and N2B3– N, higher dose + Biochar 30 t ha^− 1^

During the grain-filling period, SBE and DBE play an important role in starch synthesis [1]. In our results, SBE and DBE activity were significantly (*P* < 0.05) affected by biochar and nitrogen fertilizers (Supplementary file 2- Tables 3 and 4). The activity of SBE and DBE was low, high, moderate, and low at 7, 14, 21, and 28 DAA, respectively (Fig. 2A-D). The activity of SBE and DBE increased up to 14 DAA and gradually decreased thereafter until 28DAA. Combined biochar and N application considerably enhanced SBE and DBE activity compared with treatments in which biochar was not applied. Biochar application at a rate of 30 t ha^− 1^ combined with a low N rate (N1B3) significantly increased the activity of SBE by 35 and 59%, and DBE by 30 and 53% compared with N1B0 and N2B0, respectively. Across N rates, 20 and 30 t ha^−1^biochar increased the activity of SBE and DBE compared with treatments in which biochar was not applied. However, no significant differences in SBE and DBE activity were observed among N1B2, N2B3, and N1B3.

#### GBSS and SSS activity

**Fig. 3:**
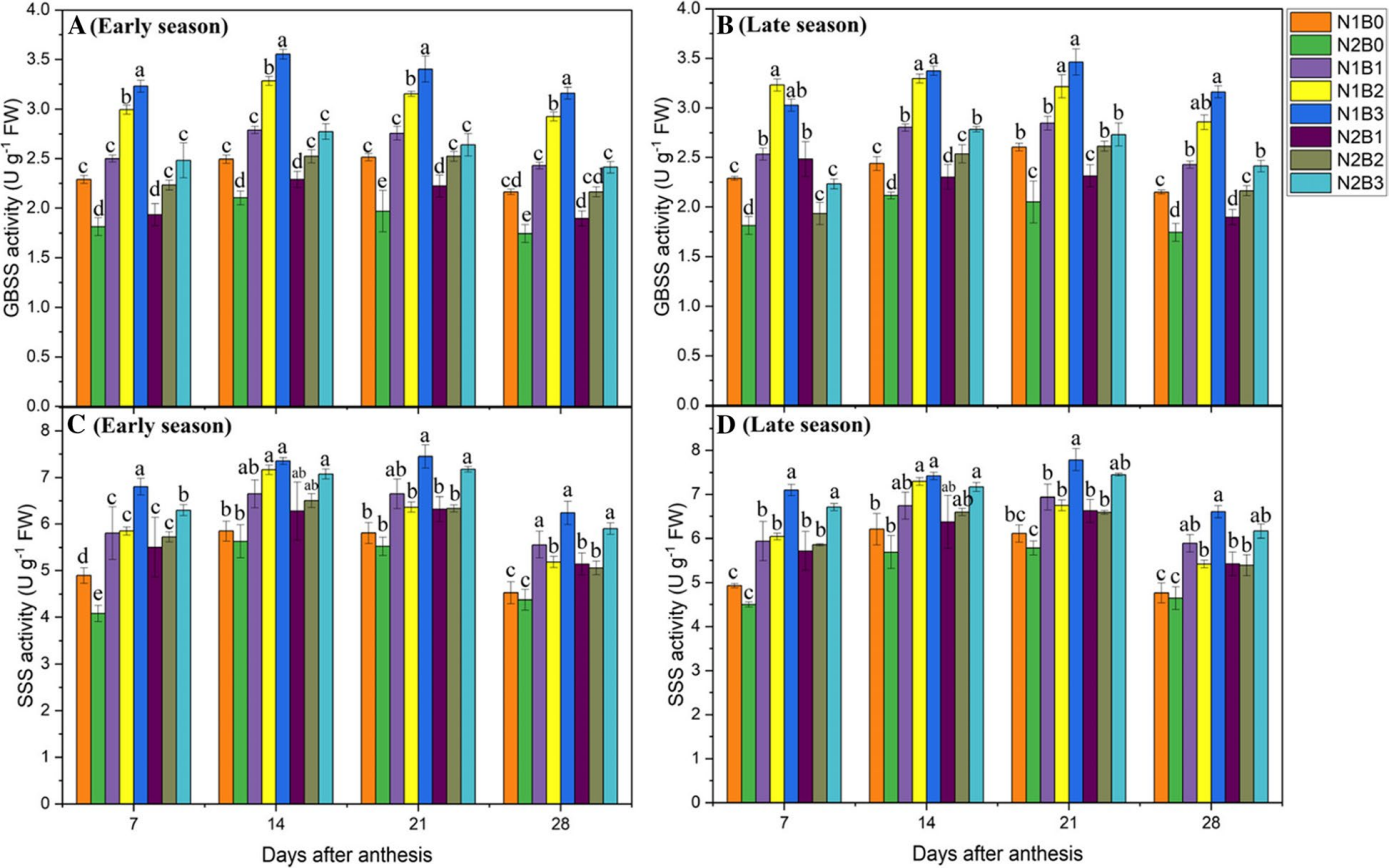
Changes in the GBSS and SSS activity 7, 14, 21, and 28 days after anthesis in response to different biochar and N levels in the early (**A**) and late (**B**) seasons. Vertical bars represent the standard error of the mean. Different letters above columns indicate significant differences (*P* < 0.05). Vertical bars represent the standard error of the mean. Note: N1B0–N, lower dose + Control (no biochar); N2B0–N, higher dose + Control (no biochar); N1B1–N, lower dose + Biochar 10 t ha^− 1^; N1B2–N,lower dose + Biochar 20 t ha^− 1^; N1B3–N, lower dose + Biochar 30 t ha^− 1^; N2B1– N, higher dose + Biochar 10 t ha^− 1^; N2B2– N, higher dose + Biochar 20 t ha^− 1^; and N2B3– N, higher dose + Biochar 30 t ha^− 1^

GBSS and SSS play a key role in the elongation of the starch molecular chain. GBSS is involved in amylose production in the rice grain. The activity of GBSS enzymes peaked at 21DAA during the grain-filling stage in both early and late seasons (Fig. 3A–D). GBSS activity was 39 and 73% higher in N1B3 compared with N1B0 and N2B0, respectively, followed by N1B2, N1B1, and N2B3 during both seasons. Lower N rates enhanced SS activity by 27% during both seasons across all biochar treatments. No significant (*P* < 0.05) difference in GBSS activity was observed between N1B2 and N1B3 (Supplementary file 2- Table 5).

Furthermore, SSS activity was also significantly (*P* < 0.05) affected by biochar amendment and nitrogen fertilizer in both seasons and regimes (Supplementary file 2-Table 6). SSS activity increased from 7 to 14DAA and then gradually decreased up to 28 DAA in both seasons (Fig. 2). Greater biochar rates under both low and high N rates increased SSS activity in all treatments and both seasons. SSS activity was 31, 11, and 24% higher in N1B3, N2B2, and N2B3 compared with N1B0 and 40, 18, and 33% higher compared with N2B0, respectively. No significant differences in SSS activity were observed among N1B2, N1B3, N2B2, and N2B3.

### Expression patterns of genes related to starch metabolism

#### Expression of SUS4 and AGPS2b

**Fig. 4:**
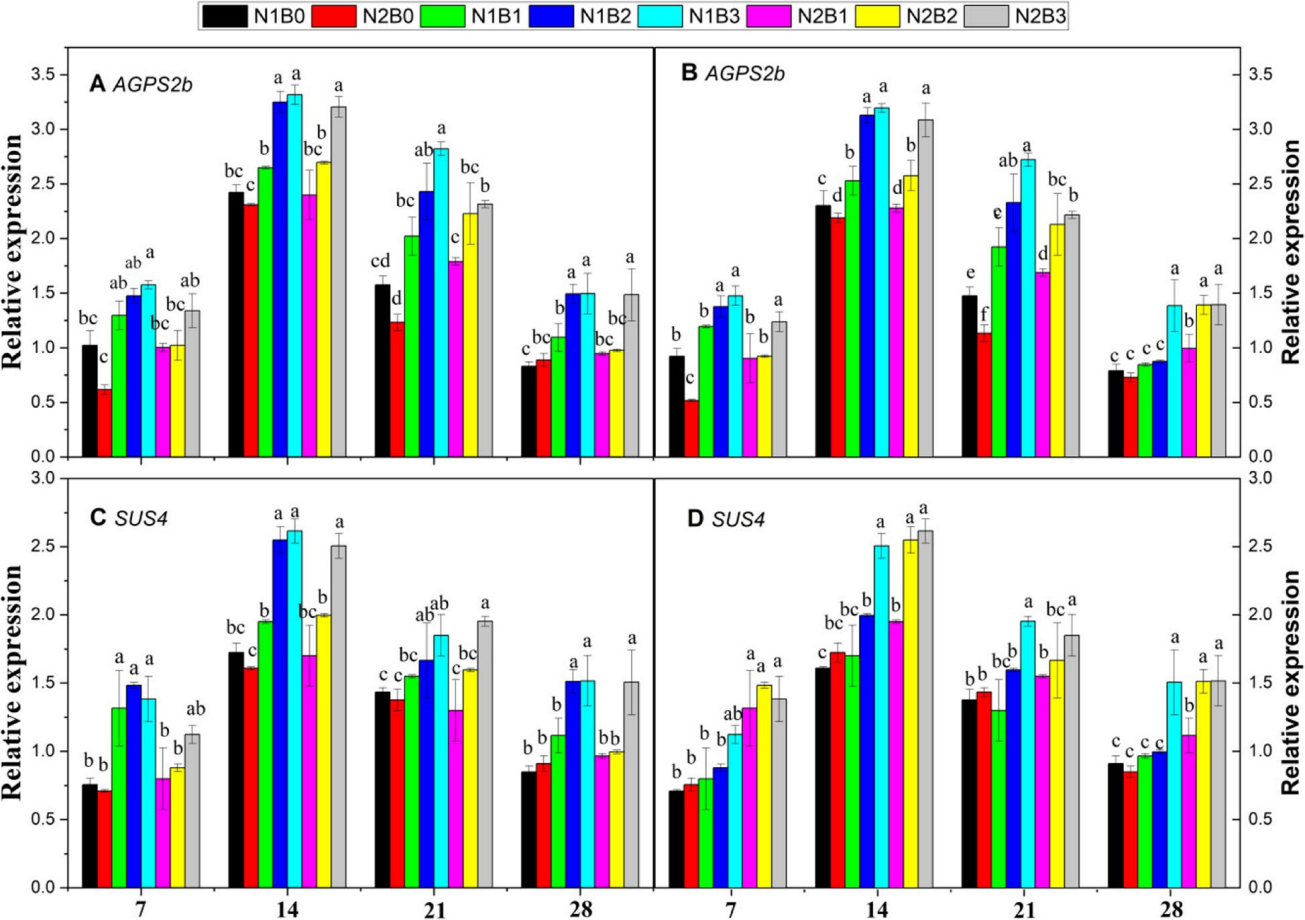
Changes in the relative expression patterns of starch metabolism-related genes [*AGPS2b* (**A**, **C**) and *SUS4* (**B**, **D**)] at 7, 14, 21, and 28 days after anthesis in response to different biochar and N levels in the early (**A**, **C**) and late (**B**, **D**) seasons. Different letters above columns indicate significant differences (*P* < 0.05). Vertical bars represent the standard error of the mean. Note: N1B0–N, lower dose + Control (no biochar); N2B0–N, higher dose + Control (no biochar); N1B1–N, lower dose + Biochar 10 t ha^− 1^; N1B2–N,lower dose + Biochar 20 t ha^− 1^; N1B3–N, lower dose + Biochar 30 t ha^− 1^; N2B1– N, higher dose + Biochar 10 t ha^− 1^; N2B2– N, higher dose + Biochar 20 t ha^− 1^; and N2B3– N, higher dose + Biochar 30 t ha^− 1^

Figure 4(A–D) shows that *SUS4* and *AGPS2b* were significantly expressed during the grain-filling phase under combined biochar and N application. Biochar application combined with a low N rate significantly increased the expression level of these genes compared with biochar application combined with a high N rate. Both genes were highly expressed at 14 DAA compared with 7, 21, and 28 DAA. The expression of *SUS4 *was 54, 35, and 53% higher in N1B3, N2B2, and N2B4 compared with N1B0 and 54, 36, and 54% higher compared with N2B0, respectively. The expression of *AGPS2b *was 58, 22, and 43% higher in N1B3, N2B2, and N2B3 compared with N1B0, respectively. Compared with N2B0, the expression of *AGPS2b *was increased by 55, 31, and 43% in N1B3, N2B2, and N2B4, respectively. Higher rates of N application decreased the expression of *AGPS2b* by 14% compared with lower N rates across all levels of biochar application. However, the expression of *SUS4* and *AGPS2 *was lower in the sole urea treatments compared with the combined biochar and N application treatments. The expression of these two genes was the highest in N1B3, followed by N1B2, N2B3, N2B2, N1B1, N2B1, N1B0, and N2B0. Generally, our results showed that biochar applied at 30 t ha^−1^combined with a low dose of N up-regulated the expression of *SUS4* and *AGPS2b* in rice under paddy field conditions

#### Expression of SSSI and ISAI

**Fig. 5:**
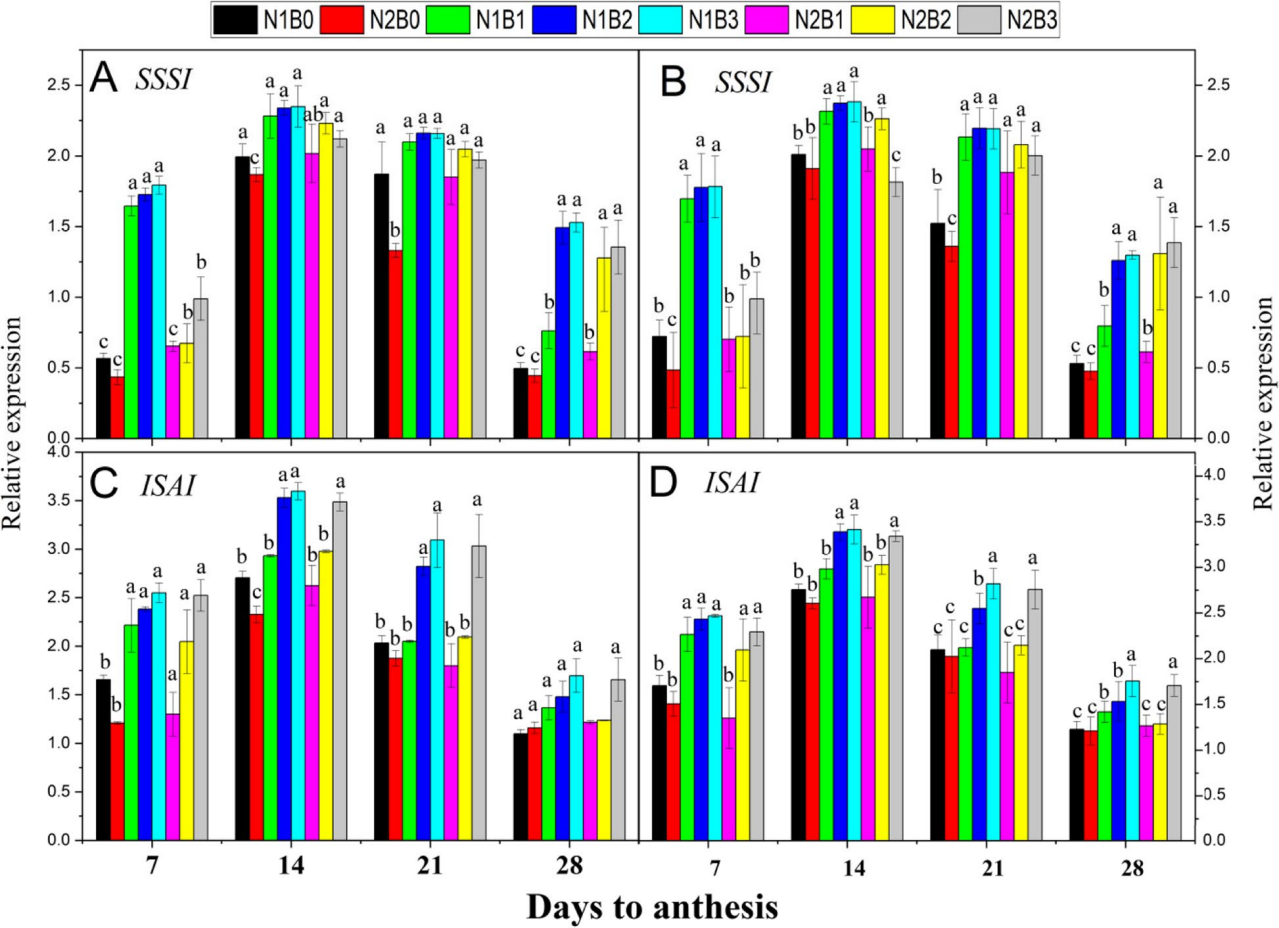
Changes in the relative expression patterns of starch metabolism-related genes [*SSSI* (**A**, **B**) and *ISAI* (**C**, **D**)] 7, 14, 21, and 28 days after anthesis in response to different levels of biochar and N fertilizers during both early (**A**, **C**) and late (**B**, **D**) seasons. Vertical bars represent the standard error of the mean. Different letters above columns indicate significant differences (*P* < 0.05).. Note: N1B0–N, lower dose + Control (no biochar); N2B0–N, higher dose + Control (no biochar); N1B1–N, lower dose + Biochar 10 t ha^− 1^; N1B2–N,lower dose + Biochar 20 t ha^− 1^; N1B3–N, lower dose + Biochar 30 t ha^− 1^; N2B1– N, higher dose + Biochar 10 t ha^− 1^; N2B2– N, higher dose + Biochar 20 t ha^− 1^; and N2B3– N, higher dose + Biochar 30 t ha^− 1^

During the grain-filling period, the expression of *SSSI* and *ISAI* was higher under biochar and N fertilizer application (Fig. 5). The patterns of *SSSI* and *ISAI* expression were the same among different biochar treatments during both seasons. The expression of *ISAI* was 44, 10, and 41% higher in N1B3, N2B2, and N2B3compared with N1B0, respectively, across seasons and treatments. Similarly, the expression of *ISAI* was increased by 59, 21, and 55% in N1B3, N2B2, and N2B3 compared with N2B0, respectively. However, the expression of *ISAI* was 13% higher under a high N rate compared with a low N rate across biochar treatments. Patterns of *SSSI* expression were the same among treatments during both seasons (Fig. 5C, and D). The expression of *SSSI* was 56, 25, and 29% higher in N1B3, N2B2, and N2B3compared with N1B0 and 73, 52, and 74% higher compared with N2B0, respectively. The expression of *SSS1* was up-regulated by 24% at a lower N rate relative to a higher N rate across different rates of biochar application.

#### Expression of GBSSI and GBSEIIb

**Fig. 6:**
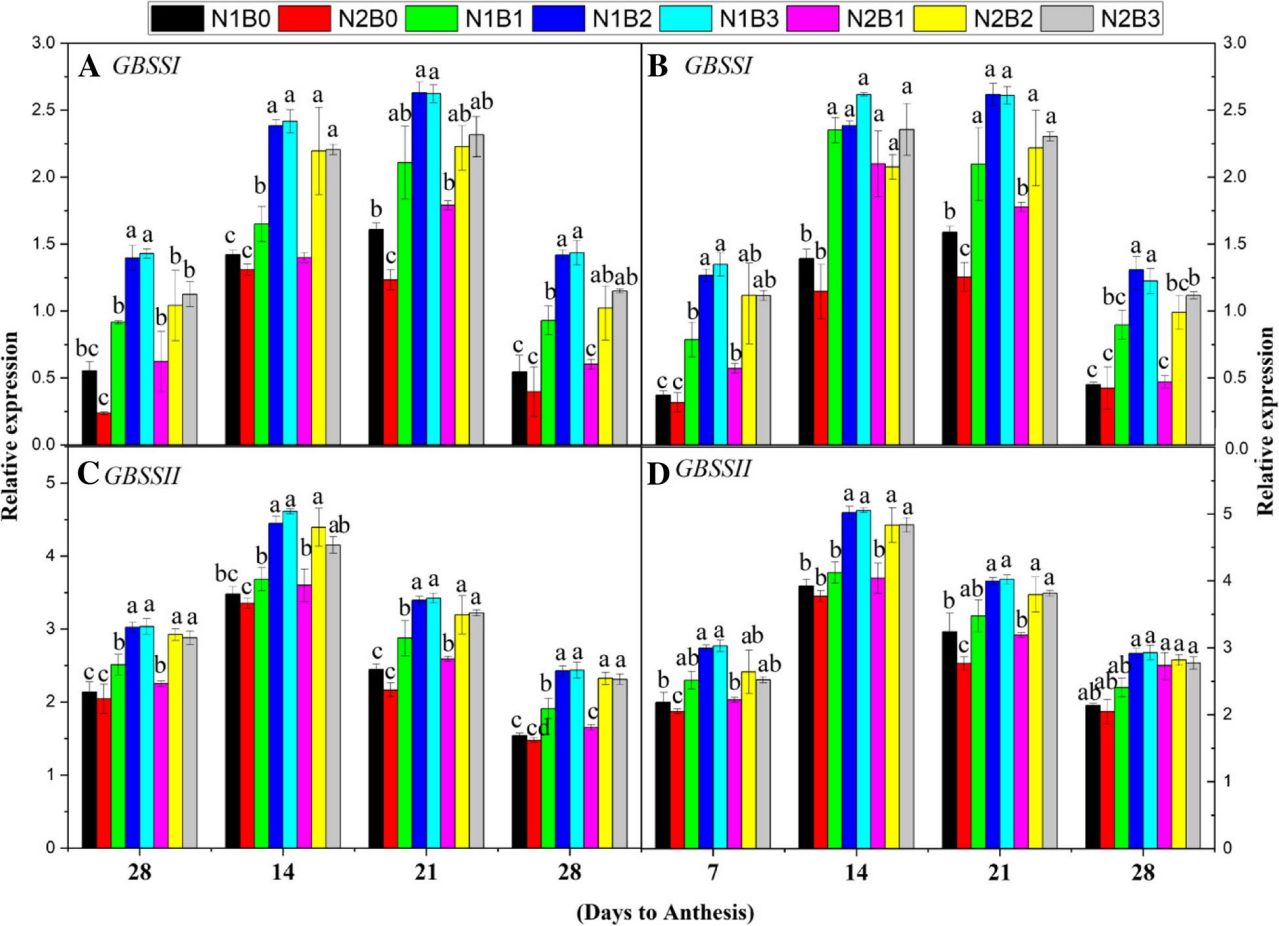
Changes in the relative expression patterns of starch metabolism-related genes [*GBSSI* (**A**, **B**) and *GBSSII* (**C**, **D**)] 7, 14, 21, and 28 days after anthesis in response to different levels of biochar and N fertilizers during the early (**A**, **C**) and late (**B**, **D**) seasons. Vertical bars represent the standard error of the mean. Different letters above columns indicate significant differences (*P* < 0.05).. Note: N1B0–N, lower dose + Control (no biochar); N2B0–N, higher dose + Control (no biochar); N1B1–N, lower dose + Biochar 10 t ha^− 1^; N1B2–N,lower dose + Biochar 20 t ha^− 1^; N1B3–N, lower dose + Biochar 30 t ha^− 1^; N2B1– N, higher dose + Biochar 10 t ha^− 1^; N2B2– N, higher dose + Biochar 20 t ha^− 1^; and N2B3– N, higher dose + Biochar 30 t ha^− 1^

The expression of *GBBSI* and *GBSEIIb* was high under biochar combined with low and high N rates during the grain-filling stage (Fig. 6A-D). The expression of G*BBSI* was up-regulated in treatments in which both biochar and N fertilizer were applied compared with sole N treatments. Across different growth stages and seasons, the expression of G*BBSI* was 95, 58, and 68% higher in N1B3, N2B2, and N2B3 compared with N1B0, respectively. The expression of *GBBSI* was up-regulated by 131, 103, and 132% in N1B2, N2B2, and N2B3 compared with N2B0, respectively. The expression of *GBSSI* was increased by 30% at the lower N rate compared with the higher N rate. The expression of *GBSEIIb* was up-regulated in N1B3, N2B2, and N2B3 by 36, 26, and 37% compared with N1B0 and by 46, 36, and 37% compared with N2B0, respectively, across DAA and seasons. Higher N rates decreased *GBSEIIb* expression by 7% compared with lower N rates. However, the expression of*ISAI* and *GBSEIIb* was down-regulated in the sole N treatments. The expression of *ISAI* and *GBSEIIb *was up-regulated by a biochar application of 30 t ha^−1^and a low N rate.

### Effect of different levels of biochar under low and high N on the rice starch content and amylose content

**Fig. 7:**
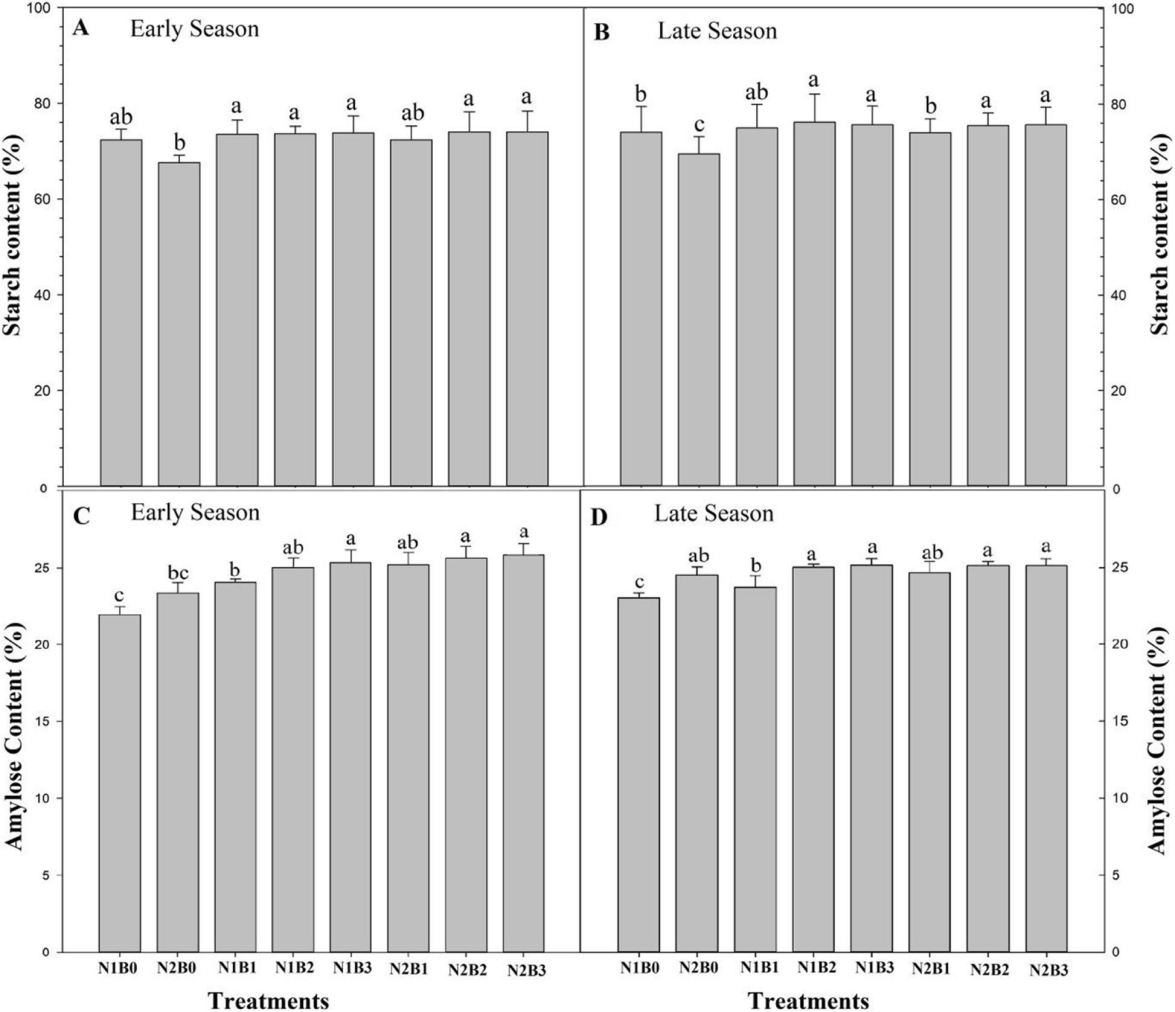
Changes in the rice grain starch (**A** and **B**) and amylose content (**C** and **D**) in response to different biochar and N levels during the early (**A**) and late (**B**) seasons. Vertical bars represent the standard error of the mean. Different letters above columns indicate significant differences (*P* < 0.05)

The starch concentration in rice grains was significantly increased under different levels of biochar combined with N fertilizer (Fig. 7). Increasing the amount of biochar applied gradually increased the starch concentration in rice grains during both seasons. The combined treatments of biochar and N (B × N) (i.e., N1B3, N2B2, and N2B3) significantly enhanced the rice grain starch content by 9.1, 9.2, and 9.3% compared with sole N application (N1B0), respectively, in both seasons. The lowest starch content in rice grain was observed in treatments in which biochar was not applied. No significant differences in the starch content between N1B3, N2B2, and N2B3 were observed.

The amylose content gradually increased as the biochar rate increased (Fig. 7). The amylose content of rice grain was increased by 12.3, 12.8, and 13.3% in N1B3, N2B2, and N2B3 compared with N1B0, respectively. Although the amylose content in rice grains was enhanced by 5, 6, and 6% in N1B3, N2B2, and N2B3 compared with N2B0, respectively, no significant differences in amylose content were observed among N1B3, N2B2, and N2B3. The lowest amylose content was observed in treatments in which biochar was not applied. A biochar rate of 30 t ha^− 1^coupled with a low N rate and a biochar rate of 20 and 30 t ha^− 1^coupled with a high N rate increased the amylose content of rice under paddy field conditions.

### Effect of different levels of biochar under low and high N on rice yield and yield components

Combined biochar and N application and season significantly affected rice yield and yield components (Table 2). Tiller’s hill^− 1^, plant height (PH), thousand-grain weight (TGW), panicle length (PL), and the grain yield (GY) of noodle rice was 62%, 4%, 19%, 23% and 30% higher in N2B3 compared with N1B0 across both seasons, respectively. Panicle hill^− 1^, PH, TGW, PL, and GY were 10.5%, 1.0%. 6.4%, 8.2%, and 5.6% higher at an N rate of 180 kg N ha^− 1^ compared with 135 kg N ha^− 1^ across biochar rates. Tiller hill^− 1^, PH, TGW, PL, and GY were higher in the early season than in the late season. Considering to the best performance of N1B2 and N1B3 on genes expression level, enzyme activities and starch content, those treatments also improved yield and yield components as compared to N1B0. However, no significant differences (*p* < 0.05) in rice yield and yield components were observed among 20 and 30 t ha^− 1^ biochar rates combined with a high N rate (N2B2 and N2B3) and 30 t ha^− 1^ biochar combined with low N (N1B3). The lowest yield and yield attributes were observed in treatments in which no biochar was applied and the N rate was low.

**Table 2:** The responses of grain yield and yield components to different levels of biochar and N fertilizers

(Kg ha^− 1^)	B	PN hill^− 1^	PH (cm)	TGW(g)	PL (cm)	GY (kg ha^−1^)
Early season						
Low N(135)	0	6.67d	114.33c	19.97d	19.94c	6122.5c
	10	9.67c	116.67b	21.97c	19.44bc	7122.5b
	20	10.13bc	117.50b	21.59c	21.05bc	7477.2ab
	30	11.0ab	118.40a	23.27ab	24.53a	7863.2a
High N (180)	0	11.33c	118.47b	23.74bc	24.50bc	7122.5b
	10	9.67c	116.95b	21.93c	21.42b	7243.5b
	20	11.33a	118.42a	23.58a	24.59a	7870.3
	30	11.33a	118.47a	23.74a	24.50a	7891.7a
Late season						
Low N(135)	0	6.48d	111.23b	19.28d	19.24b	5481.4c
	10	9.07bc	115.30a	21.28c	19.41b	6538.4c
	20	9.67a	116.53a	20.90c	20.35b	7065.5a
	30	10.33abc	116.90a	22.58ab	23.83a	7222.2a
High N (180)	0	8.67c	115.17a	21.68bc	19.31b	6481.4c
	10	9.00bc	116.12a	21.24c	19.47b	6887.4ab
	20	9.33abc	116.92a	22.89a	23.89a	7229.3a
	30	10.00ab	116.97a	23.05a	23.80a	7250.7a
SOV		PN	PH	TGW	PL	GY
Biochar (B)		**	ns	*	**	**
Nitrogen(N)		*	ns	**	*	**
Season(S)		ns	ns	ns	ns	ns
B × N		**	**	**	**	**
B × S		ns	ns	ns	ns	ns
N × S		ns	ns	ns	ns	ns

*PN* panicle number, *PH* plant height, *TGW* thousand-grain weight, *PL* panicle length, *GY* grain yield. SOV-source of variation, ** indicate the significant difference *P* ≥ 0.01 and * indicate *P* = 0.01-0.05, Values followed by the same letters within a column are not significantly different at *P* ≤ 0.05.

### Relationship between SMEs, grain starch, and amylose

**Fig. 8:**
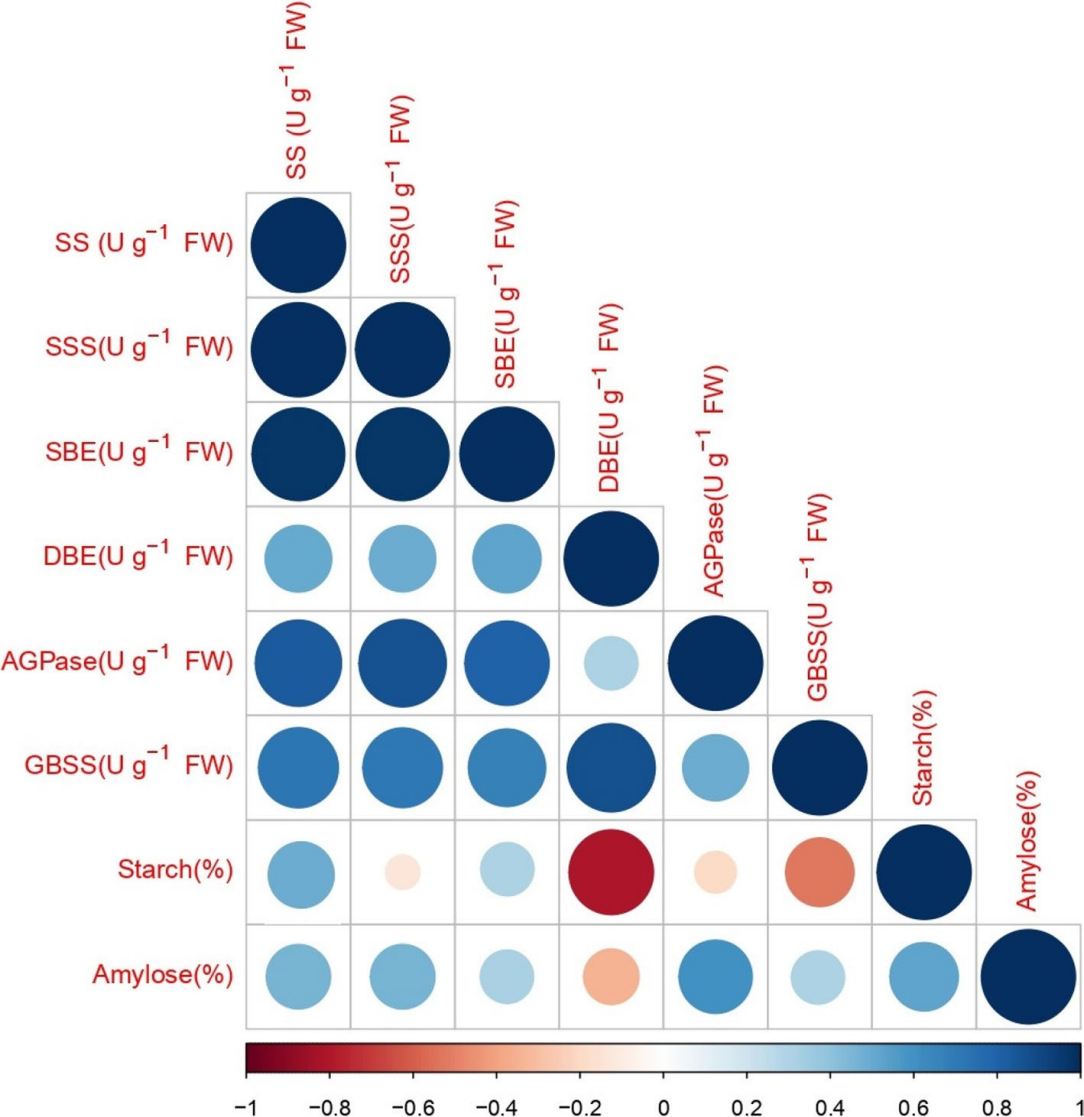
Correlation heat map of SBEs, starch content, and amylose content of rice using R software. Circle with red to dark blue color indicates the relationship from negative to positive. Circle size from small to big indicates the moderate positive/negative to strongly negative/positive relationship

SMEs is strongly associated with the starch and amylose content of rice. A heat map of the Pearson correlation coefficients confirmed that amylose content was positively correlated with SS, SSS, SBE, AGPase, and GBSS activity (Fig. 8). The activity of DBE was negatively correlated with the amylose content of rice grains. Similarly, the starch content of rice grains was highly positively correlated with SS and SBE activity, and SSS and AGPase activity was not correlated with the starch content of rice grains. The activity of DBE and GBSS was strongly negatively correlated with the starch content of rice. These findings indicate that variation in the activity of SMEs had a major effect on rice grain starch and amylose content.

## Discussion

Rice production and rice grain quality are affected by environmental factors such as geographical site, temperature, fertilizers, and soil health [1]. Several recent studies have examined the effect of biochar on rice yield and quality [16, 17]. However, few studies have examined the effects of combined biochar and N application on the activity of SMEs, the expression of related genes, and the grain starch content in noodle rice [8]. N application has previously been shown to affect starch biosynthesis by altering the activity of starch-related enzymes and the expression of genes involved in non-structural carbohydrate translocation and accumulation [35, 36].

We found that a biochar rate of 20 to 30 t ha^− 1^ combined with a low and high rate of N fertilization enhanced the accumulation of rice grain starch and the amylose content compared with sole N-treated plots. The reason for the increase in the quality of rice grain under combined biochar and N treatment might be explained by its ability to facilitate the slow release and steady accessibility of important plant nutrients during all growth stages [37], the availability of nutrients during the grain-filling stage has been shown to promote the activity of SMEs [8, 38]. Furthermore, a previous study reported that N application enhanced the accumulation of carbohydrates in plants by increasing starch and sucrose biosynthesis, the activity of related enzymes, and the transportation of related genes [39]. In addition, our study confirmed that the increase in the activity of SMEs through the addition of biochar to soil increases the amylose content and starch content of rice grains (Fig. 7). Another possible explanation for the increase in the grain quality of biochar-treated plants might be the increase in N accumulation in rice plants, which was 23–27% higher in biochar-treated plants compared with plants that were not treated with biochar [8]. In addition, the activity of SBEs during the grain milky stage at various N rates and different forms of N application can affect the amylose content and protein content [40, 41]. These results are similar to those of Fahad et al. [42], Pavlíková et al. [43], and Solaiman et al. [44] showing that the addition of biochar increases quality traits compared with control soils.

Starch synthesis in rice grain is mainly affected by SMEs during the grain milky phase [8, 45, 46]. Various SMEs such as SS, AGPase, SSS, GBSS, SBE, and DBE are involved in starch accumulation and synthesis during the maturity stage of rice [39]. A higher biochar rate and alow N rate significantly enhanced the activity of SS, AGPase, SSS, GBSS, SBE, and DBE during the grain-filling period. The increase in SMEs might stem from the increased N uptake by rice in the biochar treatments [24, 37], which enhances the uptake of nutrients by the roots and photosynthesis [37]; furthermore, N accumulation can improve the activity of starch-related enzymes [8, 17]. Biochar promoted the activity of specific enzymes, which improved the uptake of essential nutrients and the activity of SMEs [20, 47] also reported increases in enzyme activity under moderate N rates, as well as reductions in SBE activity under low N application. In addition, increases in the activity of SMEs in this study mostly stemmed from the significant up-regulation of the expression of isoform genes. Our study also confirmed that SMEs were strongly correlated to the expression of SMEs (Fig. 7). A previous study showed that lower activity of SBEs mainly stems from a decrease in the expression of SBE genes, which affects the starch accumulation in grains under high levels of N fertilizer application [20].

The expression levels of starch isoform genes, including *SSSI, ISAI*, *SUS4*, and *AGPS2b*, directly affect the starch and amylose content [7]. Biochar application at 10 to 30 t ha^− 1^ increased the expression of *SSSI, ISAI*, *SUS4*, and *AGPS2b* compared with treatments in which biochar was not applied. Our findings suggest that biochar affected the expression of genes involved in starch synthesis, increased the activity of enzymes involved in the synthesis of starch and amylose, and affected rice quality. Changes in the expression of isoform genes related to starch synthesis in response to biochar amendment might be explained by two possible mechanisms. First, biochar contains many soluble active molecules that can facilitate the formation of organic complexes, such as benzoic acid, acetoxy acid, carboxylic acid, triol, and phenolic substances [7]. The plant root system allows these active molecules to directly enter the plant. These molecules bind to specific proteins/genes engaged in starch synthesis and then activate new metabolic pathways that affect grain quality. Zhen et al. [48] and Farhangi-Abriz [49] reported that biochar amendment alters the expression levels of genes via the presence of soluble molecules in biochar that enhance salt and drought resistance. In addition, biochar is rich in silicon, which plants can uptake directly and indirectly. Several studies have suggested that silicon can improve the photosynthetic rate of plants and promote the transportation and synthesis of carbohydrates [7, 50]. Furthermore, rice grain quality, including the appearance of rice grains and the starch content, is enhanced by silicon [51, 52]. Our findings suggest that biochar combined with an appropriate rate of N fertilizer application can enhance starch metabolism, gene expression, enzyme activities, and rice grain quality.

The PH, TGW, PL, and GY of rice were significantly increased in biochar-treated plots at a low N rate compared with sole urea-treated plots (Table 2). Biochar combined with N application improves soil physiochemical properties and photosynthetic production [8, 16], thereby increasing GY and yield components. Our findings were similar to those of Ullah et al. [9] and Ndor et al. [53] showing that the addition of biochar to soil enhanced soil fertility, improved rice GY, and promoted plant N uptake. Similarly, Ali et al. [8] attributed the increase in GY and yield components in biochar-treated soil to higher soil microbial biomass, carbon, N, and chlorophyll. Numerous researchers have confirmed the positive effects of biochar since the start of the twenty-first century. Our study showed that combined biochar and N application improves the activity of SMEs and the expression of related genes in rice under paddy field conditions.

## Conclusion

The combined application of biochar and N fertilizer can promote the activity of SMEs by affecting the expression of starch-related genes, which in turn affects important traits determining the quality of noodle rice, such as the amylose content and starch content. Our study also confirmed that the activity of GBSS and SBE was positively correlated with the amylose and starch content of rice grains. Therefore, the optimal treatment for maximizing GY, grain quality, and the grain-filling rate was a biochar rate of 20 to 30 t ha^− 1^ combined with an N rate of 135 kg ha^− 1^.

## Supplementary Information

**Additional file 1: Table 1.** Primers for each gene used in this experiment

**Additional file 2: Table 1.** Analysis of Variance table for SSS enzyme activity. **Table 2.** Analysis of Variance table for AGPase enzyme activity. **Table 3.** Analysis of Variance table for SBE enzyme activity. **Table 4.** Analysis of Variance table for DBE enzyme activity. **Table 5.** Analysis of Variance table for GBSS enzyme activity. Table 6. Analysis of Variance table for SS enzyme activity

## Data Availability

The datasets used and/or analyzed during the current study available from the corresponding author on reasonable request.

## Abbreviations

B: Biochar
N: Nitrogen
t: Ton
SMEs: Starch metabolism enzymes
AGPase: ADP-glucose pyrophosphorylase
GBSS: Granule-bound starch synthase
SBE: Starch branching enzyme
DBE: Starch debranching enzyme
SSS: Soluble starch synthase
SS: Starch synthase (SS)
PH: Plant height
TGW: Thousand-grain weight
PL: Panicle length
GY: Grain yield
