# Supplementary material for: Combined application of biochar and nitrogen fertilizer promotes the activity of starch metabolism enzymes and the expression of related genes in rice in a dual cropping system
Source: BMC Plant Biol. 2021 Dec 18;21:600. doi: 10.1186/s12870-021-03384-w (PMC8684189; doi:10.1186/s12870-021-03384-w)
Supplement: Supplementary file 1 — Additional file 1: Table 1. Primers for each gene used in this experiment [file 12870_2021_3384_MOESM1_ESM.docx]

| Genes | Accession No. | Primers | Sequences(5′-3′) | Amplified fragment length(bp) |
| --- | --- | --- | --- | --- |
| *actin* | LOC-4333919 | Forward | CCACTATGTTCCCTGGCATT | 178 |
|  |  | Reverse | GTACTCAGCCTTGGCAATCC |  |
| *SBEIIb* | LOC-Os02g32660.1 | Forward | GCCGCAGGAGAAATCCCATA | 150 |
|  |  | Reverse | GTTGATCTTTGGCTCCGTGC |  |
| *GBSSI* | LOC-Os06g04200.3 | Forward | GGGGAAAGACCGGTGAGAAG | 236 |
|  |  | Reverse | GATGCCATTGGGCTGGTAGT |  |
| *ISA1* | LOC-Os08g40930.1 | Forward | GCTGGTGGTTTCGCTGAATG | 203 |
|  |  | Reverse | CCACAGTTCCAGCTGAGGTT |  |
| *SSSI* | LOC-Os06g06560.1 | Forward | CCAGTCTTGTGCCAGTCCTT | 220 |
|  |  | Reverse | TTGACTGCCTCACCCTTGTC |  |
| *AGPS2b* | LOC-Os08g25734.2 | Forward | TCTTGACCGCAGTGTCGATG | 228 |
|  |  | Reverse | GCTCTTGACAGGTGACGGTT |  |
| *SUS4* | LOC-Os03g22120.1 | Forward | GGCCAGTACAACGATCCGTA | 198 |
|  |  | Reverse | ATGACCCTTATGCCGATGCC |  |

**Table 1.** Primers for each gene used in this experiment.
