# Supplementary material for: Combined application of biochar and nitrogen fertilizer promotes the activity of starch metabolism enzymes and the expression of related genes in rice in a dual cropping system
Source: BMC Plant Biol. 2021 Dec 18;21:600. doi: 10.1186/s12870-021-03384-w (PMC8684189; doi:10.1186/s12870-021-03384-w)
Supplement: Supplementary file 2 — Additional file 2: Table 1. Analysis of Variance table for SSS enzyme activity. Table 2. Analysis of Variance table for AGPase enzyme activity. Table 3. Analysis of Variance table for SBE enzyme activity. Table 4. Analysis of Variance table for DBE enzyme activity. Table 5. Analysis of Variance table for GBSS enzyme activity. Table 6. Analysis of Variance table for SS enzyme activity [file 12870_2021_3384_MOESM2_ESM.docx]

**Abbreviations:**

**T=Treatments; S=Seasons, Days= Days after anthesis**

**Table 1. Analysis of Variance table for SSS enzyme activity**

**Source DF SS MS F P**

Days 3 56.097 18.6989 97.86 0.0000

S 1 2.676 2.6757 14.00 0.0003

T 7 75.888 10.8411 56.74 0.0000

Days*S 3 0.240 0.0799 0.42 0.7403

Days*T 21 4.626 0.2203 1.15 0.3042

S*T 7 0.021 0.0030 0.02 1.0000

Days*S*T 21 0.326 0.0155 0.08 1.0000

Error 128 24.458 0.1911

Total 191 164.331

Grand Mean 6.0909 CV 7.18

Statistix 8.1 11/4/2021, 9:52:56 AM

**LSD All-Pairwise Comparisons Test of SSS for T**

**T Mean Homogeneous Groups**

T3 7.0982 A

T8 6.7456 B

T5 6.2741 C

T2 6.2607 C

T7 6.0082 D

T6 5.9231 D

T1 5.3889 E

T4 5.0286 F

Alpha 0.05 Standard Error for Comparison 0.1262

Critical T Value 1.979 Critical Value for Comparison 0.2497

Error term used: Error, 128 DF

There are 6 groups (A, B, etc.) in which the means

are not significantly different from one another.

**LSD All-Pairwise Comparisons Test of SSS for S**

**S Mean Homogeneous Groups**

2 6.2090 A

1 5.9729 B

Alpha 0.05 Standard Error for Comparison 0.0631

Critical T Value 1.979 Critical Value for Comparison 0.1248

Error term used: Error, 128 DF

All 2 means are significantly different from one another.

**Table 2. Analysis of Variance table for AGPase enzyme activity**

**Source DF SS MS F P**

T 7 1.40845 0.20121 73.37 0.0000

S 1 0.01847 0.01847 6.74 0.0105

Days 3 1.00900 0.33633 122.64 0.0000

T*S 7 0.00225 0.00032 0.12 0.9971

T*Days 21 0.06279 0.00299 1.09 0.3665

S*Days 3 0.00048 0.00016 0.06 0.9814

T*S*Days 21 0.00078 0.00004 0.01 1.0000

Error 128 0.35103 0.00274

Total 191 2.85325

Grand Mean 0.5481 CV 9.56

Grand Mean 0.5481 CV 9.56

**LSD All-Pairwise Comparisons Test of data for T**

**T Mean Homogeneous Groups**

T8 0.6504 A

T4 0.6412 A

T7 0.6218 A

T3 0.5847 B

T6 0.5198 C

T5 0.4894 D

T2 0.4865 D

T1 0.3908 E

Alpha 0.05 Standard Error for Comparison 0.0151

Critical T Value 1.979 Critical Value for Comparison 0.0299

Error term used: Error, 128 DF

There are 5 groups (A, B, etc.) in which the means

are not significantly different from one another.

Statistix 8.1 11/4/2021, 7:03:59 AM

**Table 3. Analysis of Variance table for SBE enzyme activity**

**Source DF SS MS F P**

T 7 53.777 7.6824 184.45 0.0000

Days 3 141.878 47.2925 1135.48 0.0000

S 1 8.074 8.0737 193.85 0.0000

T*Days 21 0.786 0.0374 0.90 0.5927

T*S 7 0.024 0.0034 0.08 0.9991

Days*S 3 1.214 0.4048 9.72 0.0000

T*Days*S 21 0.044 0.0021 0.05 1.0000

Error 128 5.331 0.0416

Total 191 211.128

Grand Mean 3.0914 CV 6.60

Statistix 8.1 11/4/2021, 7:04:22 AM

**LSD All-Pairwise Comparisons Test of SBE for T**

**T Mean Homogeneous Groups**

T7 3.6818 A

T4 3.6752 A

T8 3.5671 A

T3 3.3872 B

T5 2.9946 C

T1 2.7116 D

T6 2.4061 E

T2 2.3077 E

Alpha 0.05 Standard Error for Comparison 0.0589

Critical T Value 1.979 Critical Value for Comparison 0.1166

Error term used: Error, 128 DF

There are 5 groups (A, B, etc.) in which the means

are not significantly different from one another.

**LSD All-Pairwise Comparisons Test of SBE for S**

**S Mean Homogeneous Groups**

2 3.2965 A

1 2.8863 B

Alpha 0.05 Standard Error for Comparison 0.0295

Critical T Value 1.979 Critical Value for Comparison 0.0583

Error term used: Error, 128 DF

All 2 means are significantly different from one another.

**Table 4. Analysis of Variance table for DBE enzyme activity**

**Source DF SS MS F P**

T 7 5.7409 0.82013 45.12 0.0000

Days 3 3.1562 1.05208 57.88 0.0000

S 1 0.5039 0.50393 27.72 0.0000

T*Days 21 2.1987 0.10470 5.76 0.0000

T*S 7 0.3851 0.05502 3.03 0.0056

Days*S 3 0.0675 0.02250 1.24 0.2988

T*Days*S 21 1.4527 0.06918 3.81 0.0000

Error 128 2.3267 0.01818

Total 191 15.8318

Grand Mean 1.4100 CV 9.56

Statistix 8.1 11/4/2021, 6:54:29 AM

**LSD All-Pairwise Comparisons Test of DBE for T**

**T Mean Homogeneous Groups**

T4 1.7869 A

T3 1.5325 B

T5 1.4275 C

T1 1.3797 CD

T7 1.3660 CD

T6 1.3165 D

T8 1.3036 D

T2 1.1673 E

Alpha 0.05 Standard Error for Comparison 0.0389

Critical T Value 1.979 Critical Value for Comparison 0.0770

Error term used: Error, 128 DF

There are 5 groups (A, B, etc.) in which the means

are not significantly different from one another.

**Table 5. Analysis of Variance table for GBSS enzyme activity**

**Source DF SS MS F P**

T 7 36.8013 5.25733 248.79 0.0000

Days 3 4.7932 1.59773 75.61 0.0000

S 1 0.0200 0.01997 0.95 0.3328

T*Days 21 0.6974 0.03321 1.57 0.0662

T*S 7 0.2156 0.03080 1.46 0.1883

Days*S 3 0.0638 0.02128 1.01 0.3921

T*Days*S 21 0.6384 0.03040 1.44 0.1123

Error 128 2.7048 0.02113

Total 191 45.9344

Grand Mean 2.5512 CV 5.70

Statistix 8.1 11/4/2021, 6:58:55 AM

**LSD All-Pairwise Comparisons Test of GBSS for T**

**T Mean Homogeneous Groups**

T4 3.3124 A

T3 3.1186 B

T5 2.6281 C

T8 2.5593 C

T1 2.3679 D

T7 2.3361 D

T6 2.1676 E

T2 1.9194 F

Alpha 0.05 Standard Error for Comparison 0.0420

Critical T Value 1.979 Critical Value for Comparison 0.0830

Error term used: Error, 128 DF

There are 6 groups (A, B, etc.) in which the means

are not significantly different from one another.

**LSD All-Pairwise Comparisons Test of GBSS for S**

**S Mean Homogeneous Groups**

2 2.5614 A

1 2.5410 A

Alpha 0.05 Standard Error for Comparison 0.0210

Critical T Value 1.979 Critical Value for Comparison 0.0415

Error term used: Error, 128 DF

There are no significant pairwise differences among the means.

**Table 6. Analysis of Variance table for SS enzyme activity**

**Source DF SS MS F P**

T 7 2886490 412356 206.53 0.0000

Days 3 1804621 601540 301.28 0.0000

S 1 662583 662583 331.85 0.0000

T*Days 21 316794 15085 7.56 0.0000

T*S 7 10938 1563 0.78 0.6031

Days*S 3 34631 11544 5.78 0.0010

T*Days*S 21 31993 1523 0.76 0.7588

Error 128 255568 1997

Total 191 6003617

Grand Mean 513.99 CV 8.69

Statistix 8.1 11/4/2021, 9:48:11 AM

**LSD All-Pairwise Comparisons Test of SS for T**

**T Mean Homogeneous Groups**

T2 632.20 A

T7 630.21 A

T3 628.66 A

T8 622.76 A

T5 491.94 B

T6 402.65 C

T1 385.44 C

T4 318.03 D

Alpha 0.05 Standard Error for Comparison 12.899

Critical T Value 1.979 Critical Value for Comparison 25.523

Error term used: Error, 128 DF

There are 4 groups (A, B, etc.) in which the means

are not significantly different from one another.

**LSD All-Pairwise Comparisons Test of SS for S**

**S Mean Homogeneous Groups**

2 572.73 A

1 455.24 B

Alpha 0.05 Standard Error for Comparison 6.4495

Critical T Value 1.979 Critical Value for Comparison 12.761

Error term used: Error, 128 DF

All 2 means are significantly different from one another.

Statistix 8.1 11/4/2021, 9:52:29 AM
